# Genetic Characterization of Carbapenem-Resistant *Klebsiella pneumoniae* Clinical Isolates in a Tertiary Hospital in Greece, 2018–2022

**DOI:** 10.3390/antibiotics12060976

**Published:** 2023-05-28

**Authors:** Charalampos Zarras, Theodoros Karampatakis, Styliani Pappa, Elias Iosifidis, Eleni Vagdatli, Emmanuel Roilides, Anna Papa

**Affiliations:** 1 Department of Microbiology, Medical Faculty, School of Health Sciences, Aristotle University of Thessaloniki, 541 24 Thessaloniki, Greece; zarraschak6@gmail.com (C.Z.); s_pappa@hotmail.com (S.P.); annap@auth.gr (A.P.); 2 Microbiology Department, Hippokration General Hospital, 546 42 Thessaloniki, Greece; evagdatli@gmail.com; 3 Infectious Disease Unit, 3rd Department of Pediatrics, Medical Faculty, School of Health Sciences, Hippokration General Hospital, 546 42 Thessaloniki, Greece; iosifidish@gmail.com (E.I.); roilides@auth.gr (E.R.)

**Keywords:** carbapenem-resistant *Klebsiella pneumoniae*, whole genome sequencing, core genome single-nucleotide polymorphism analysis, molecular epidemiology

## Abstract

Background: Carbapenem-resistant *Klebsiella pneumoniae* (CRKP) is a serious public health issue. The study aimed to identify the antimicrobial resistance and accessory genes, the clonal relatedness, and the evolutionary dynamics of selected CRKP isolates recovered in an adult and pediatric intensive care unit of a tertiary hospital in Greece. Methods: Twenty-four CRKP isolates recovered during 2018–2022 were included in the study. Next-generation sequencing was performed using the Ion Torrent PGM Platform. The identification of the plasmid content, MLST, and antimicrobial resistance genes, as well as the comparison of multiple genome alignments and the identification of core genome single-nucleotide polymorphism sites, were performed using various bioinformatics software. Results: The isolates belonged to eight sequence types: 11, 15, 30, 35, 39, 307, 323, and 512. A variety of carbapenemases (KPC, VIM, NDM, and OXA-48) and resistance genes were detected. CRKP strains shared visually common genomic regions with the reference strain (NTUH-K2044). ST15, ST323, ST39, and ST11 CRKP isolates presented on average 17, 6, 16, and 866 recombined SNPs, respectively. All isolates belonging to ST15, ST323, and ST39 were classified into distinct phylogenetic branches, while ST11 isolates were assigned to a two-subclade branch. For large CRKP sets, the phylogeny seems to change approximately every seven SNPs. Conclusions: The current study provides insight into the genetic characterization of CRKP isolates in the ICUs of a tertiary hospital. Our results indicate clonal dispersion of ST15, ST323, and ST39 and highly diverged ST11 isolates.

## 1. Introduction

*Klebsiella pneumoniae* is recognized as an important Gram-negative opportunistic pathogen that causes community- and hospital-acquired infections globally [1]. It can cause ventilator-associated pneumonia among patients in intensive care units (ICUs), bloodstream infections, and urinary tract infections [2,3,4]. The emergence of multidrug-resistant (MDR) *K. pneumoniae* has become a major public health problem, causing a significant increase in morbidity and mortality worldwide [5]. The spread of carbapenem-resistant *K. pneumoniae* (CRKP) has generally been considered an increasingly serious issue for clinical practice due to the limitation of therapeutic options [6,7,8]. Greece is endemic for CRKP [9,10,11,12,13]; data from the European Antimicrobial Resistance Surveillance Network committee for the year 2020 indicate that the prevalence of CRKP in Greece was 66.3%, the highest among the European countries [14].

The rapid dissemination of CRKP is closely related to the antimicrobial resistance genes carried by plasmids and transferable genetic elements [15,16,17,18]. CRKP strains accumulate antimicrobial resistance genes due to inadequate implementation of infection control measures in healthcare settings and the irrational use of antimicrobials, resulting in the emergence of MDR, extremely drug-resistant, and pan-drug-resistant phenotypes [19]. The main mechanism of resistance to carbapenems is through the production of enzymes called carbapenemases, with *K. pneumoniae* carbapenemase (KPC) being the most prevalent, followed by Verona integron-encoded metallo-β-lactamase (VIM), New Delhi metallo-β-lactamase (NDM), and OXA-48 carbapenemases [20,21,22]. The contribution of outer membrane protein (Omp) deficiencies, such as *OmpK35*, *OmpK36,* and *OmpK37,* is considered a secondary mechanism in the emergence of CRKP [23,24,25]. The pathogenic potential of these strains is determined by virulence factors such as capsular polysaccharide synthesis, fimbriae, pili, outer membrane proteins, and iron acquisition systems [26].

The application of next-generation sequencing (NGS) has become a powerful tool for obtaining whole genome sequences (WGS), resulting in genetic characterization of the strains and a better understanding of the genomic diversity, providing fast and in-depth information about bacterial pathogenicity [27]. WGS analysis is recommended for genotyping and determining relatedness between clinical *K. pneumoniae* strains, as well as for the surveillance of virulence genes [28]. The aim of the present study was to identify the antimicrobial resistance and accessory genes of selected CRKP isolates recovered in an adult intensive care unit (ICU) and a pediatric ICU (PICU) of a tertiary hospital in Greece, to evaluate their clonal relatedness through core genome single-nucleotide polymorphism (cgSNP) analysis, and to calculate the evolutionary dynamics of this bacterial population.

## 2. Results

### 2.1. Antimicrobial Susceptibility Testing 

**Figure 1:**
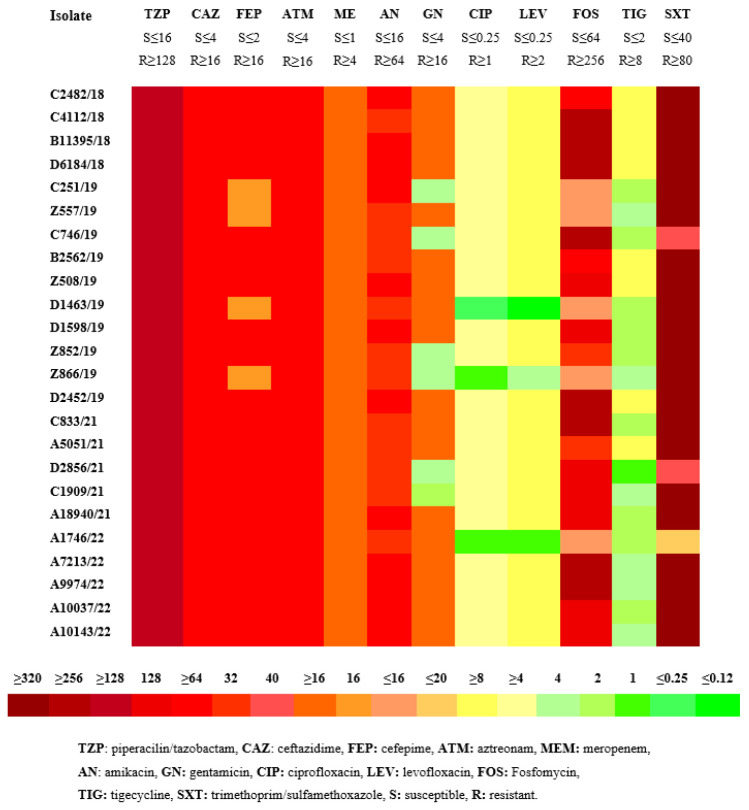
MICs (μg/mL) heatmap of the 24 clinical isolates of carbapenem-resistant *K. pneumoniae* (CRKP) according to CLSI.

All CRKP isolates were resistant to β-lactam antibiotics (including penicillins, cephalosporins, monobactams, and carbapenems). Nineteen isolates (79.2%) were resistant to at least one aminoglycoside, 23 isolates (95.8%) showed resistance to quinolones, eight isolates (33.3%) were resistant to fosfomycin and tigecycline, and 21 (87.5%) to cotrimoxazole. Four out of five isolates that carried the class A β-lactamase *bla*_KPC_ showed susceptibility to ceftazidime-avibactam (CAZ-AVI). The phenotypic resistance rates to antimicrobials of CRKP isolates are shown in the heatmap in Figure 1.

### 2.2. MLST, Antimicrobial Resistance Genes, Plasmids

The CRKP isolates belonged to eight different STs: ST11 (7, 29.1%), ST39 (7, 29.1%), ST323 (3, 12.5%), ST15 (7, 29.1%), ST307 (1, 4.2%), ST30 (1, 4.2%), ST512 (1, 4.2%), and ST35 (1, 4.2%).

The resistance genes and the plasmid content per isolate are detailed in Table 1. The prevalence of genes conferring resistance to beta-lactams was 100%, to aminoglycosides 79.2%, to quinolones 95.8%, to fosfomycin and tigecycline 33.3%, and to cotrimoxazole 87.5%. The most prevalent plasmid was IncF, which is associated with the spread of several extended-spectrum β-lactamase (ESBL) genes or carbapenemases and was detected in all CRKP strains; additional plasmids were found in fifteen strains, such as Col and IncC.

### 2.3. Virulence Factors and Efflux and Regulator Systems 

The virulence factors detected included fimbrial genes, as 23/24 isolates possessed the *mrk* cluster (type 3 fimbriae); siderophores, as yersiniabactin genes (*ybt*, *irp,* and *fyu*A) were present in 17 isolates; and genes encoding aerobactin siderophore (*iut*) were identified in five isolates (three of them also carried yersiniabactin genes). In addition, the *kfu* gene (*Klebsiella* ferric iron uptake, which is a regulator of the iron transport system) was detected in four isolates. 

Efflux and regulator system genes encoding the AcrAB efflux pump (*acr*, *mar*, *sox*, *rob*, *ram*, *sdi*A, *fis, and env*R) or the OqxAB efflux pump (*oqx* and *rar*) were present in all isolates (Table 1).

### 2.4. Genomic Comparison among CRKP Strains

**Figure 2:**
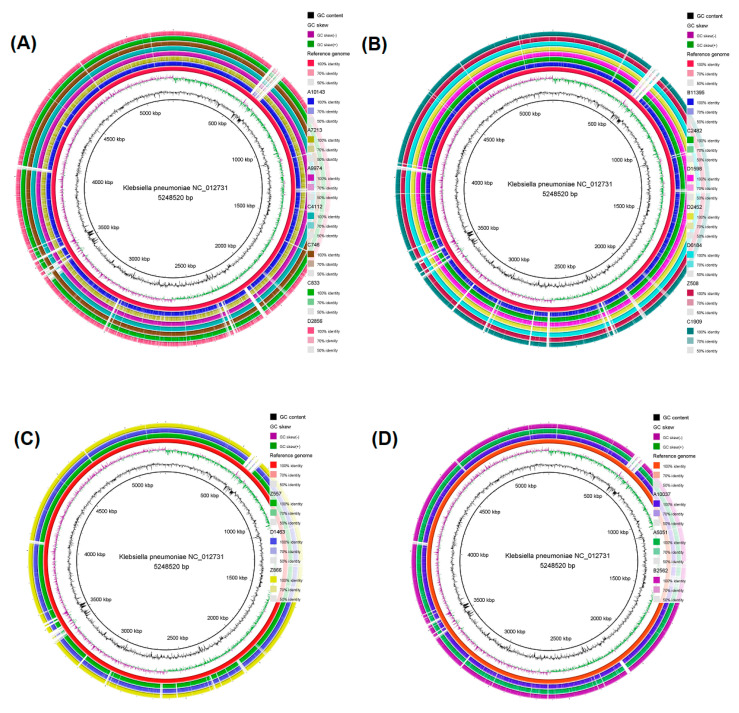
Circular genome map of (**A**) seven ST11 CRKP strains, (**B**) the seven ST39 CRKP strains, (**C**) the three ST323 CRKP strains, and (**D**) the three ST15 CRKP strains. The genome maps were produced through the BLAST Ring Image Generator (BRIG) software. The genomes of the seven ST11 CRKP isolates (A10143, A7213, A9974, C4112, C746, C833, and D2856), the seven ST39 CRKP isolates (B11395, C2482, D1598, D2452, D6184, Z508 and C1909), the three ST323 CRKP strains (Z557, D1463, and Z866) and the three ST15 CRKP strains (A10037, A5051, and B2562) were mapped separately to the reference genome *K. pneumoniae* NC_012731. From the inner to the external ring, the innermost ring displays the size of the genome in kbp, followed by GC content (black), GC skew (dark green and dark purple), and *K. pneumoniae* NC_012731 (red). The white vertical gaps represent sequences of the *K. pneumoniae* NC_012731 reference strain that are absent in the sequences of the ST11, ST39, ST323, and ST15 CRKP strains or present sequences <50% identical to the reference genome.

Circular genome maps were generated for the four STs with more than one isolate (ST11, ST39, ST323, and ST15). It was found that isolates belonging to the same ST shared many identical regions with the reference strain. CRKP isolates C4112 and C746 of the ST11, D2452, and D6184 of the ST39, Z866 of the ST323, and B2562 of the ST15 displayed visually the highest similarity with the reference strain throughout their whole genome (Figure 2).

### 2.5. CgSNP-Based Phylogenetic Analysis

The length of the multiple core genome alignments of all CRKP isolates was 1,892,845 base pairs. The core genome length corresponds to about 36.1% of the reference genome length. Nearly 1.9 million columns in the core genome alignment (almost 98.5%) showed identical nucleotides for all isolates, meaning that 1.5% of the genome is polymorphic. 

**Figure 3:**
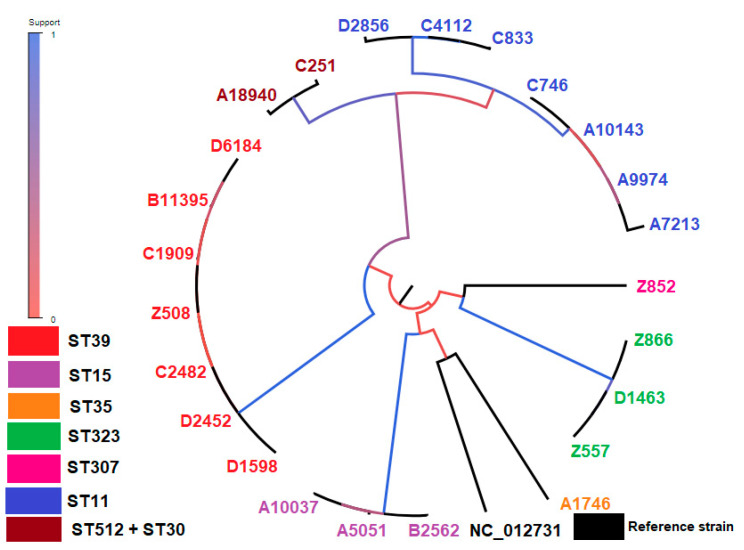
The tree built on the entire core genome with PhyML. The branch color indicates the fraction of supporting versus supporting plus clashing SNPs for each branch of the core tree, which supports the initial bipartition of the strains. MLST types: **ST11:** D2856, C4112, C833, C746, A10143, A9974, A7213 (blue color), **ST512:** A18940 (brown color), **ST30:** C251 (brown color), **ST307:** Z852 (pink color), **ST323:** Z866, D1463, Z557 (green color), **ST35:** A1746 (orange color), **ST15:** B2562, A5051, A10037 (purple color), **ST39:** D1598, D2452, C2482, Z508, C1909, B11395, D6184 (red color).

A phylogenetic tree including the cgSNP-based phylogenetic analysis of all isolates, along with the reference strain, is seen in Figure 3.

When performing all possible pairwise analyses among ST15 isolates, an average of 17 recombined SNPs (range 5–26) were detected; the SNP diversity along the core genome was very low, that is, 0–3 cgSNPs per kilobase. ST323 CRKP strains presented an average of six recombined SNPs (range 5–6), and the SNP diversity was extremely low, that is, 0-2 cgSNPs per kilobase. ST39 CRKP strains displayed an average of 16 recombined SNPs (range 4–43), with the SNP diversity being low, that is, 0–5 cgSNPs per kilobase. Finally, ST11 CRKP strains showed an average of 866 recombined SNPs (range 1–1652), with the SNP diversity being clearly higher, that is, 0–66 cgSNPs per kilobase.

**Figure 4:**
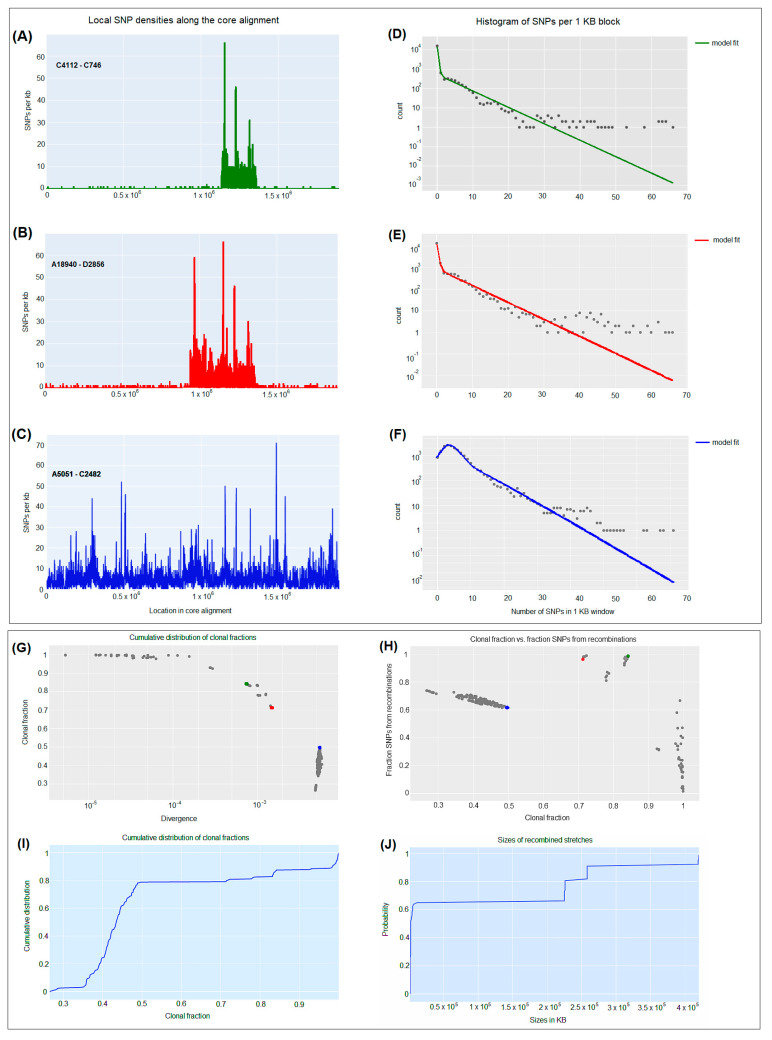
Pairwise analysis of recombination between carbapenem-resistant *K. pneumoniae* CRKP strains. (**A**–**C**) SNP densities (SNPs per kilobase) along the core genome for three pairs of CRKP strains with various nucleotide diversities. (**D**–**F**) Corresponding histograms for the number of SNPs per kilobase (dots) together with fits of the Poisson mixture model for C4112-C746 (green), A18940-D2856 (red), and A5051-C2482 (blue) CRKP strains. The vertical axis is on a logarithmic scale. (**G**) For each pair of CRKP strains (dots), the fraction of the genome that was inherited clonally is shown as a function of the nucleotide divergence of the pair, shown on a logarithmic scale. The three CRKP pairs that are highlighted in panels (**A**–**F**) are depicted as green, red, and blue dots. (**H**) Fraction of all SNPs that lie in recombined regions as a function of the clonally inherited fraction of the genome. (**I**) Cumulative distribution of the clonal fractions of CRKP pairs. (**J**) Cumulative distribution of the lengths of recombined segments for CRKP pairs that are in the mostly clonal regime.

For pairs of CRKP isolates, for instance, C4112 and C746 with relatively low diversity (7.5 × 10^−4^) (Figure 4G), the impact of recombination is almost directly evident in the pattern of SNP density along the genome. While the SNP density is very low along most of the genome, that is, 0–2 SNPs per kilobase, there are a few segments where the SNP density is much higher (Figure 4A) and comparable to the typical SNP density between randomly selected highly diverged CRKP isolates, that is, 30–65 SNPs per kilobase (Figure 4C). For some pairs of isolates, for example, A18940 and D2856, with higher genetic diversity (1.5 × 10^−3^) (Figure 4G), the frequency of these high-spiking recombined regions increases (Figure 4B), until most of the genome is covered by such regions when comparing highly divergent isolates (5.5 × 10^−3^), for instance, A5051 and C2482 (Figure 4C,G). The distribution of SNP densities was described by a majority of clonally inherited regions with mostly up to three SNPs per kilobase and a long tail of recombined regions with up to 50 or 65 SNPs per kilobase (Figure 4D–F).

**Figure 5:**
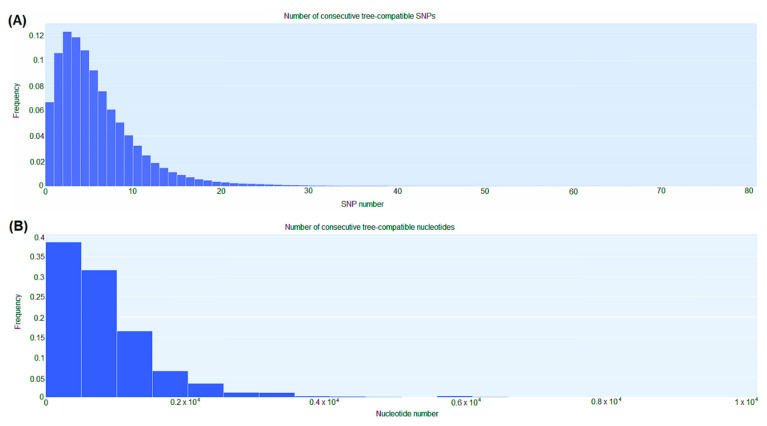
SNP compatibility along the core genome alignment displays the shortness of tree-compatible segments. (**A**) Probability distribution of the number of consecutive SNP columns that are consistent with a common phylogeny for the core genome alignment. (**B**) Probability distribution of the number of consecutive nucleotides consistent with a common phylogeny for the core genome alignment.

Figure 5 highlights the distribution of the lengths of tree-compatible stretches. This distribution had a mode at *n* = 2, and stretches were normally around 7–12 consecutive SNPs and very rarely longer than 15–20 consecutive SNPs (Figure 5A). Similarly, tree-compatible segments were typically just a few hundreds of base pairs long (around 1000–1500 base pairs) and very rarely more than 2000 base pairs (Figure 5B).

**Figure 6:**
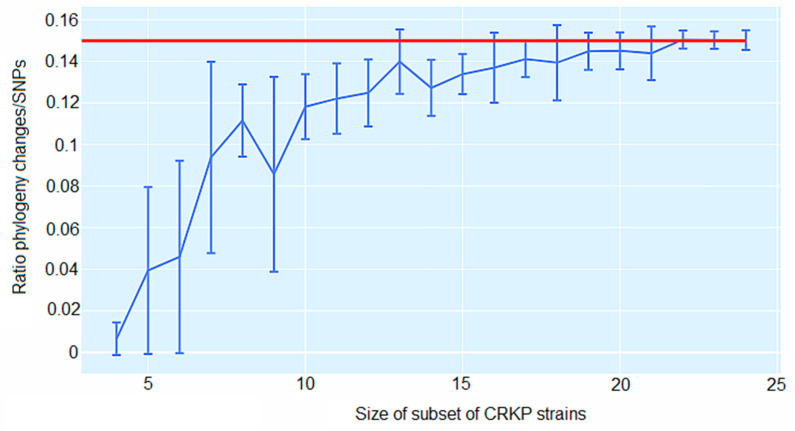
Ratio C/S of the minimal number of phylogeny changes C to substitutions S for random subsets of carbapenem-resistant *K. pneumoniae* (CRKP) strains using the alignment from which 5% of potentially homoplastic positions have been removed. For CRKP strain numbers ranging from *n* = 4 to *n* = 24, random subsets of n strains were collected, and the ratios C/S of phylogeny changes to SNPs in the alignment were calculated. The figure shows box-whisker plots that indicate, for each CRKP strain number *n*, means, and standard deviations for C/S across subsets. The horizontal red line displays C/S = 0.15.

Figure 6 underscores the ratio C/S for random subsets of all 24 isolates. When comprising all 24 isolates, a ratio C/S = 0.15 was obtained for the 5% homoplasy-corrected full alignment. For small subsets of isolates, the C/S ratio displayed significant fluctuations. For instance, for subsets of *n* = 7 isolates, the C/S ratio presented a mean of 0.094 ± 0.046. However, as the number of isolates in the subset increased, the ratios converged to the value C/S = 0.15, and for large subsets of isolates, there was little variation in this ratio. Consequently, for alignments of large sets of isolates, the phylogeny should change at least approximately every seven SNPs (Figure 6). In addition, on average, each randomly chosen position on the core genome has been overwritten at least *T* = 4344 times (5% homoplasies removed).

## 3. Materials and Methods 

### 3.1. Clinical Isolates-Setting

The present study included 24 CRKP isolates collected from June 2018 to July 2022 from 18 patients hospitalized in the ICU (8 females and 10 males) and six patients hospitalized in the PICU (4 females and 2 males) of a tertiary university-affiliated general hospital in Greece. The median age of the adult patients was 57.5 years (range 30–88), while the median age of the children was 6.2 years (range 0.25–17). The selection of the isolates was based on the type of carbapenemase(s) previously detected in order to include all possible combinations, as they were pre-screened as previously described [11]. Eight isolates were collected from colonization sites (rectal swabs), while 16 isolates were taken from various types of clinical samples (Table 2).

### 3.2. Ethics Approval

The study was approved by the Ethics Committee of Aristotle’s University Medical Faculty (no. of approval 5.160/18/12/2019).

### 3.3. Microbiological Methods-Antimicrobial Susceptibility Testing

CRKP isolates were identified through the automated system VITEK 2 (bioMérieux, Marcy-l’Étoile, France) using the Gram-negative identification card (GN ID). Antimicrobial susceptibility testing (AST) of isolates was performed using the AST318 and XN10 cards, while the use of minimum inhibitory concentration (MIC) test strips (Liofilchem srl, Roseto, Italy) was applied to confirm susceptibility to ceftazidime-avibactam (CAZ-AVI). Identification and susceptibility cards were interpreted according to the manufacturer’s instructions. The Clinical and Laboratory Standards Institute (CLSI) susceptibility breakpoints were applied for the interpretation of results [29]. The breakpoints approved by the US Food and Drug Administration (FDA) were used to interpret the MICs of tigecycline [30].

All CRKP isolates were previously screened for carbapenemase production using a lateral flow immunochromatographic assay (LFIA) (NG-Test CARBA 5, NG Biotech, Paris, France). The results were confirmed by multiplex PCR in a single reaction following a modified protocol that was previously described [11,31].

### 3.4. DNA Extraction and Whole Genome Sequencing

Bacterial DNA was extracted using the DNA extraction kit (Qiagen, Hilden, Germany). The DNA concentration (sample starting concentration between 10–100 ng/μL) was measured using the Qubit double-strand DNA HS assay kit (Q32851, Life Technologies Corporation, Grand Island, NY, USA). 

NGS was performed using the Ion Torrent PGM Platform (Life Technologies Corporation). All procedures regarding purification, ligation, barcoding, library preparation, emulsion PCR, and enrichment were performed following the manufacturer’s instructions. PCR products were loaded on an Ion-316™ chip. The Ion PGM Hi-Q View (200) chemistry was applied using the Ion PGM Hi-Q view sequencing kit (A25592). 

### 3.5. Assembly Assessment and Genome Annotation

The software Geneious Prime version 2021.2.1 and BLAST Ring Image Generator (BRIG) were used to produce genome assemblies and annotation data [32,33]. *K. pneumoniae* NTUH-K2044 strain (GenBank accession number NC_012731) was used as a reference sequence, which is an invasive and hypervirulent *K. pneumoniae* strain with the hypervirulent plasmid pK2044 [34].

### 3.6. MLST and Detection of Antimicrobial Resistance Genes and Plasmids

The sequence types (ST), plasmid types, and antimicrobial resistance genes were identified through the Center for Genomic Epidemiology website using the MLST web server and the related online databases, PlasmidFinder version 2.0 and Resfinder version 4.1, respectively [35,36,37]. The Comprehensive Antibiotic Resistance Database was also used, with the selection criteria for hits set to perfect (100%) and strict (>95%) identity to the reference sequence [38]. The detection of genes related to virulence capsule, efflux, and regulator systems was performed according to the protocols available from the Pasteur Institute (https://bigsdb.pasteur.fr/cgi-bin/bigsdb/bigsdb.pl?db=pubmlst_klebsiella_seqdef (accessed on 20 October 2022).

### 3.7. Genomic Comparison–Core Genome Single-Nucleotide Polymorphism (cgSNP)-Based Phylogenetic Analysis

The BRIG software was used to visually compare multiple genome alignments using a default minimum threshold of 50% [33]. The reference sequence alignment-based phylogeny builder version 1.12 (REALPHY; https://realphy.unibas.ch/realphy/ (accessed on 15 November 2022) was used to identify relevant SNP sites for core genome phylogenetic analysis [39]. Default input parameters were applied for cgSNP identification. The *K. pneumoniae* NTUH-K2044 sequence was used as a reference for the multiple alignments and the visual comparison of the whole genomes [34]. The produced alignment was used for the creation of an unrooted tree on the entire core genome through the approximate maximum likelihood (ML) method using PhyML [40]. Pairwise analysis between isolates was also performed using REALPHY, as previously described by Dixit et al. [41]. The calculation of SNPs between isolates was conducted through REALPHY, using a Poisson mixture model plus a negative binomial for the recombined regions. REALPHY was also used to assess to what extent mutually consistent SNPs are clustered along the alignment; the lengths of segments along the alignment that are consistent with a single phylogeny were calculated [39]. In addition, the lower bound for the ratio between the total number of phylogeny changes (C) and substitution events (S) (C/S) that occur along the core genome alignment was calculated. The same ratio was calculated for any subset of CRKP isolates by removing 5% of potentially homoplastic sites [42]. Moreover, a rough estimate for the average number of times *T* that a randomly chosen position in the core genome alignment has been overwritten by recombination in its history was calculated, that is, the time since the genetic ancestors of the position in the alignment diverged from a common ancestor.

## 4. Discussion

In the current study, the WGSs of 24 CRKP isolates were analyzed. It was found that they belonged to eight different STs, with ST11 and ST39 being the most prevalent. Ten out of 24 isolates (41.6%) carried *bla*_NDM-1,_ and two of them (8.3%) transferred concurrently *bla*_NDM-1_ and *bla*_OXA-48_. Six out of 10 NDM-producers (60.0%) were assigned to ST11, which is common in NDM-positive CRKP strains and is globally distributed, especially in Asia and particularly in China, but also in Europe [43,44]. In Greece, the first outbreak of an NDM-1-producing *K. pneumoniae* ST11 clonal strain occurred during 2011–2012 [44], while the first report of a *K. pneumoniae* ST11 clinical isolate co-producing NDM-1 and OXA-48 carbapenemases was reported in 2016 [22]. NDM-1-producing ST11 CRKP strains have been isolated during the same period in other Balkan countries [45]. However, three out of 10 NDM producers (30.0%) were assigned to ST15; this has also been previously described [46].

ST39 was also the most prevalent type, encompassing seven isolates (29.1%); two of them carried *bla*_KPC-2_, while one of them (14.3%) harbored *bla*_KPC-33_ (a KPC-2 variant). CRKP strains belonging to ST39 have been detected only in sporadic cases so far, like ST39 carrying *bla*_KPC-2_ [47] or *bla*_VIM-1_ [48]. ST39 isolates transferring KPC-33 have been recently detected in Greece [8]. The present study also included the recently reported four ST39 isolates carrying both *bla*_KPC-2_ and *bla*_VIM-1_ [21]. Recently, Kuzina et al. highlighted the concurrent isolation of *bla*_NDM-1_, *bla*_KPC-2_, and *bla*_OXA-48_ in ST39 CRKP isolates [49]. Three CRKP isolates (12.5%) were assigned to ST323; sporadic cases of ST323 CRKP have been previously reported in Greece and other European countries [50,51]. Another study from Tunisia reports the isolation of clonally related ST323 CRKP isolates [52]. It is of interest that one isolate, A1746/22, carried the VEB-25 gene (article submitted for publication); VEB-25 is a variant of VEB-1 and confers resistance to CAZ-AVI [53,54].

Twenty of the 24 isolates harbored at least two genes encoding aminoglycoside-modifying enzymes (AMEs), and six of them carried one 16S rRNA methylase gene (*arm*A, *rmt*B). In addition, 12 of these isolates carried at least one gene of the three AME classes (acetyltransferases-*aac,* phosphotransferase-*aph*, nucleotransferases-*ant*). A disagreement between phenotype and genotype has been detected in five isolates (20.8%) (detection of AME-encoding resistance genes but phenotypically sensitive to both aminoglycosides tested). A disagreement of approximately 10% between genotype and phenotype for aminoglycosides for the VITEK 2 automated system has been reported previously [55].

Fluoroquinolone resistance was observed in seven isolates (29.2%), which carried the plasmid-encoded *qnr* gene, as previously described for CRKP isolates [56]. The multidrug efflux pump oqxAB gene was detected in all strains [57], while the *aac(6′)-Ib-cr* gene (which encodes an aminoglycoside acetyltransferase that modifies not only aminoglycosides but also fluoroquinolones) has been identified in 16 isolates, as previously described [56].

For fosfomycin resistance, the gene *fos*A was detected in all isolates, but 16 of them (66.7%) were phenotypically susceptible, evidenced by an increase in the MIC, as previously described [58]. Resistance genes for cotrimoxazole (*sul* or *dfr*A) were found in all isolates except one. The Inc-type plasmids detected in all isolates and the Col-type plasmids detected in 15 isolates have been reported in other studies [21,59].

In terms of adhesive structures, almost all isolates possessed the *mrk* cluster, characteristic of type 3 fimbriae, which promotes bacterial adhesion to both abiotic and biotic surfaces [60]. Regarding siderophores, the majority of isolates carried yersiniabactin genes that are significantly associated with pathogenesis and invasive infections in humans [61], while aerobactin (detected in five isolates) is more specific for hypervirulent *K. pneumoniae* [62]. The acquisition of a chimeric plasmid including genes for the siderophores yersiniabactin and aerobactin grants cefiderocol activity against CRKP strains [63].

NGS allows for more accurate analyses regarding the clonal relatedness of strains compared to conventional typing methods [64]. Several studies have described the molecular epidemiology of CRKP isolates through NGS using a gene-by-gene comparison or SNP analysis [65,66]. 

In pairwise comparisons, isolates of ST15, ST323, and ST39 presented an average of 17, 6, and 16 recombined SNPs, respectively, implying the clonal relatedness of these isolates in accordance with their ST classification. Similarly, Miro et al. report the clonal relatedness of OXA-48-producing ST405 CRKP strains further studied with core genome MLST, showing between 6 and 17 cgSNPs [64]. Moreover, Onori et al. reveal the clonal relatedness of sporadic KPC-producing ST258 CRKP strains, presenting an average of 20 or 27 cgSNPs [67]. ST15 and ST39 strains display a higher number of cgSNPs compared to ST323 strains, and this is highlighted as a slightly increased diversity in the corresponding clades of the phylogenetic tree (Figure 3). However, ST15 and ST39 has been isolated over a 33- and 36-month period, respectively, while ST323 have been collected over a 2.5-month period (Table 2). This could indicate a difference in the measured paces of the molecular clock between these clades, as previously proposed [67].

On the contrary, ST11 isolates showed an average of 866 cgSNPs, indicating the polyclonality of these isolates. This indication is further strengthened by the variability in their content of carbapenemase and antimicrobial resistance genes (Table 1) and by their phylogenetic division into two distinct subclades (Figure 3). The high average of cgSNPs occurred between ST11 strains belonging to these two different subclades, resulting in SNP patterns (Figure 4A). Most probably, these high SNP density patterns result from horizontal recombination events, e.g., the transfer of a plasmid or phage, as previously proposed for other bacterial species [42]. These results underscore the higher discriminatory power of cgSNP analysis compared to MLST [64].

In addition, the current study highlighted novel ways in which these cgSNPs could be used to quantify phylogenetic structures and the role of recombination in genome evolution. It was shown that there are pairs of closely related isolates and that most of their DNA has been clonally inherited; however, most of the changes are derived from recombination events. When focusing on the core genome alignment, it has been shown that, until the current genetic state of CRKP strains, each genomic locus has been overwritten thousands of times by recombination in the history of their evolution. Sakoparnig et al. reached the same conclusions when focusing on *E. coli* isolates, and they disclosed a similar C/S ratio [42]. Moreover, an attempt was made to calculate the probability for a pair of SNPs to be consistent with a common phylogeny as a function of their genome distance [68]. Several other studies have also attempted to quantify recombination in bacteria and assess the relative rate with which different lineages have recombined, focusing on different types of bacteria [42,68]. To the best of our knowledge, our study is the first to accomplish such a task by studying CRKP strains.

One of the basic challenges among WGS-based typing methods is the definition of a cut-off value for the identification of the clonal relatedness between isolates; however, this remains doubtful, as it seems to be not only species-dependent but also population-dependent [64,67,69]. Thus, a threshold range should be evaluated along with clinical epidemiological data. 

In conclusion, the current study provides insight into the genetic characterization of CRKP isolates circulating during a 4-year period in the ICUs of a Greek tertiary hospital. Phylogenetic analysis showed that some of them were epidemiologically related. Given that NGS has become more affordable, its use in hospital settings is extremely helpful, as the obtained data could guide infection control and prevention strategies.

## Figures and Tables

**Table 1:** Genetic characteristics of the 24 carbapenem-resistant *K. pneumoniae* isolates of the study.

Isolates	ST	Plasmids	Antibiotic Resistance Genes Affecting					Virulence	Efflux/Regulator	Capsule
			Other Beta-Lactams	Carba-Penems	Amino-Glycosides	Quinolones	Other			
C2482/18	39	ColRNAI IncA/C2 IncFIB IncFIB(pQil) Col (pHAD28)	*bla*_SHV-79_ *bla*_TEM-1B_	*bla*_KPC-2_ *bla*_VIM-1_	*aac(3)-Iid* *aac(6′)-Ib* *aac(6′)-Im* *aant(2″)-Ia* *aph(2″)-Ib* *aph(3* *′* *)-Ia*	*oqxA,* *oqxB*	*fosA, sul1, Sul3, dfrA1*	*fyuA,* *mrkABCDFHIJ ybtASTX*	*acrB, marAR, soxSR, ramA, rob sdiA,* fis, *envR, oqxBR, rarA*	*wzc,*
C4112/18	11	ColRNAI IncFIA(HI1) IncFIB(K) IncFII(K)	*bla*_CTX-M-15_ *bla*_SHV-11_	*bla*_NDM-1_	*aac(3)-Iia, aac(6′)-Ib aph(3′)-Ia*	*aac(6′)-lb-cr, oqxA, oqxB*	*fosA, sul2* *dfrA14, catB3,* *tet(D)*	*iutA,* *mrkABCDFHIJ*	*acrABR marAR soxSR ramAR rob sdiA, fis, envR, oqxR,* r*arA*	*wzc, wzi*
B11395/18	39	IncFIB (AP001918) IncFIB(pQil) IncFII(K)	*bla*_SHV-79_ *bla*_TEM-1B_	*bla*_KPC-2_	*aac(3)-Iid, aadA1, aadA2, aph(3* *′* *)-Ia*	*oqxA, oqxB*	*fosA, sul2,* *sul3, dfrA12,* *cmlA1*	*fyuA, mrkABCDFHIJ, ybtEQSTU*	*acrABR marAR soxSR, ramA, sdiA, fis, envR, oqxA, rarA*	*wzc, wzi*
D6184/18	39	ColRNAI IncFIB (AP001918) IncFIB(pQil)	*bla*_SHV-11_ *bla*_SHV-40_ *bla*_ΤΕΜ-1Β_	*bla*_KPC-2_	*aac(3)-Iid,* *aac(6′)-Ib,* *aph(3′)-Ia,* *aadA1, aadA2*	*aac(6′)-lb-cr, oqxA, oqxB*	*fosA, sul2,* *sul3, tet(M), dfrA12, catA1, atB3,*	*fyuA, irp1, irp2, mrkABCDFHIJ, ybtAEPQSTUX*	*acrABR marAR* *oxSR, ramA, rob, sdiA, fis, envR, qxABR, rarA*	*wzc, wzi*
C251/19	30	ColRNAI IncA/C2 IncFIB(K) IncFII(K) IncX3	*bla*_SHV-12,_ *bla*_TEM-1A_ *bla*_OXA-9_	*bla*_KPC-2_ *bla*_VIM-1_	*aac(6* *′* *)-Ib aph(3* *′* *)-Ia* *aadA2 aadA15*	*qnrS1, aac(6′)-lb-cr, oqxA, oqxB*	*fosA, sul1,* *dfrA1, dfrA12, catA, tet(D)*	*fyuA, irp1, iutA mrkBCDFIJ, ybtAEPQSTU*	*acrABR, marAR, soxSR, ramAR, rob, sdiA, envR, oqxABR, rarA*	*wzc, wzi*
Ζ557/19	323	IncFIB(K) IncFIB(pQil) IncFII(K) IncFIB(Mar) IncFIB (pKPHS1) IncHI1B	*bla*_SHV-1_ *bla*_SHV-99_	*bla*_KPC-2_ *bla*_VIM-1_	*ant(2″)-Ia,* *aac(6′)-Il,* *aadA1*	*qnrA1* *oqxA, oqxB*	*fosA, fosA7, sul1, dfrA1*	*mrkABCDFIJ*	*acrAR, marA, soxSR, ramA, rob, sdiA, fis, envR, oqxA, rarA*	*wzc, wzi*
C746/19	11	ColRNAI IncFIB(K) IncFIB(pQil) IncFII(K), IncR	*bla*_SHV-1_ *bla*_OXA-1_	*bla*_KPC-2_	*aac(6′)-lb-cr* *aph(3′)-Ia aadA2*	*aac(6′)-lb-cr,* *oqxA* *oqxB*	*fosA, sul1*	*fyuA, irp1, irp2 iutA, mrkABCDFIJ, ybtAEUX*	*acrAB, marAR, soxSR, ramAR, rob, sdiA, fis*, *envR, oqxABR,*	*wzi*
B2562/19	15	IncFIB(K) IncFII(K) IncFIA(HI1)	*bla*_CTX-M-15_ *bla*_TEM-1B_ *bla*_OXA-1_	*bla*_NDM-1_	*aac(3)-Iia* *aac(6* *′* *)-Ib3* *aph(3* *″* *)-Ib* *aph(6)-Id*	*aac(6′)-lb-cr, oqxA, oqxB*	*fosA, sul2, dfrA14, catB3*	*fyuA, irp1, kfuABC, mrkABCDFIJ, ybtAPQSTUX*	*acrABR, marAR, soxSR, ramA, rob, sdiA,* fis, *envR, rarA*	*wzi*
Ζ508/19	39	ColRNAI IncA/C2 IncFIB IncFIB(pQil)	*bla*_SHV-11_ *bla*_SHV-40_ *bla*_ΤΕΜ-1Β_ *bla*_OXA-116_ *bla*_OXA-366_	*bla*_KPC-2_ *bla*_VIM-1_	*aac(3)-Iid* *aac(6′)-Ib3 aac(6′)-Im aph(2″)-Ib* *aph(3′)-Ia* *aph(3′)-Via* *aadA24*, a*rmA, aac(6’)-Il*	*qnrS1 aac(6′)-lb-cr, oqxA,* *oqxB*	*fosA, sul1, sul3, frA1,* *tet(M)*	*fyuA, irp1, irp2, mrkABCDFHIJ, ybtAEPQSTUX*	*acrBR, marAR, soxSR, ramA, rob, sdiA,* fis, *envR, oqxABR, rarA*	*wzc, wzi*
D1463/19	323	IncFIB(K) IncFIB(Mar) IncFIB (pKPHS1) IncFIB(pQil) IncFII(K) IncHI1B	*bla*_SHV-99_	*bla*_KPC-2_ *bla*_VIM-1_	*ant(2″)-Ia* *aac(6* *′* *)-Il*	*oqxA* *oqxB*	*fosA, fosA7, dfrA1*	*mrkABCDFHJ,*	*acrAR, marA, soxSR, ramA, rob, sdiA,* fis, *envR, oqxA,* *rarA*	*wzc, wzi*
D1598/19	39	ColRNAI IncA/C2 IncFIB IncFIB(pQil)	*bla*_SHV-40_ *bla*_TEM-1B_	*bla*_KPC-2_ *bla*_VIM-1_	*aac(3)-Iid* *aac(6* *′* *)-Il* *aac(6* *′* *)-Im* *ant(3)-Ia* *aph(2* *″* *)-Ib* *aph(3* *′* *)-Ia*	*qnrS1, aac(6* *′* *)-lb-cr, oqxA* *oqxB*	*fosA, sul1, sul3, dfrA1*	ND	ND	*wzi*
Z852/19	307	IncA/C2 IncFIB(pQil)	*bla*_SHV-28_	*bla*_KPC-2_ *bla*_VIM-1_	*aac(6* *′* *)-Il* *aadA24*	*oqxA* *oqxB*	*fosA, sul1, dfrA1, frA14*	*fyuA, irp1, irp2 iutA, mrkABCDFHIJ, ybtAEPQSTX*	*acrABR, marAR, soxSR, ramAR, rob, sdiA,* fis, *envR, oqxABR, rarA*	*wzc,* *wzi*
Z866/19	323	IncFIB(K) IncFIB(Mar) IncFIB (pKPHS1) IncFIB(pQil) IncFII(K) IncHI1B	*bla*_SHV-99_	*bla*_KPC-2_ *bla*_VIM-1_	*aadA1*	*oqxA* *oqxB*	*fosA, fosA7,* *Sul1*	*mrkABCDFHIJ*	*acrAR, marA, soxSR, ramA, rob, sdiA,* fis, *envR, oqxAR, rarA*	*wzc,* *wzi*
D2452/19	39	ColRNAI IncA/C2 IncFIB(pQil)	*bla*_SHV-79_ *bla*_TEM-1B_	*bla*_KPC-2_ *bla*_VIM-1_	*aac(3)-Iid* *aac(6′)-Il* *aac(6* *′* *)-Im* *ant(3)-Ia* *aph(2* *″* *)-Ib* *aph(3* *′* *)-Ia*	*qnrS1, aac(6* *′* *)-lb-cr,* *oqxA* *oqxB*	*fosA, sul1, Sul4, dfrA1*	*fyuA, irp1, irp2 mrkABCDFHIJ, ybtAEPQSTX*	*acrBR, marAR, soxSR, ramA, rob, sdiA,* fis, *envR, oqxABR, rarA*	*wzc,* *wzi*
C833/21	11	Col(pHAD2) IncFIA(HI1) IncFIB(K) IncFII(K)	*bla*_SHV-182_	*bla*_OXA-48_ *bla*_NDM-1_	*aph(3′)-Ia* *aac(6′)-lb-cr*	*aac(6′)-lb-cr, oqxA,* *oqxB*	*fosA, sul2,* *dfrA14, catB3*	*mrkABCDFHIJ*	*acrABR, marA, soxSR, ramAR, rob, sdiA, fis, envR, rarA*	*wzc,* *wzi*
A5051/21	15	IncFIA(HI1) IncFIB(K) IncFII(K)	*bla*_SHV-28_ *bla*_SHV-106_	*bla*_NDM-1_	*aac(6′)-lb-cr*	*aac(6′)-lb-cr* *oqxA*	*fosA* *catB3*	*fyuA, kfuC, mrkABCDFHIJ* *ybtQU*	*acrABR, marA, soxSR, ramAR, rob, sdiA, fis, envR, rarA*	*wzcwzi*
D2856/21	11	Col440II IncFIA(HI1) IncFIB(K) IncFII(K) IncR	*bla*_SHV-182_	*bla*_OXA-48_ *bla*_NDM-1_	*aph(3′)-Ia* *aac(6′)-lb-cr*	*oqxA*	*fosA, sul2,* *dfrA14, catB3, tet(A)*	*mrkABCDJ,* *ybtAPQS*	*acrABR, marAR, soxSR, ramAR, rob, sdiA, fis, envR, oqxAR, rarA*	*wzcwzi*
C1909/21	39	ColRNAI IncFII(K)	*bla*_SHV-40, -79, -85, -89_	*bla*_KPC-33_	*aac(6* *′* *)-Ib* *aac(6* *′* *)-lb-cr*	*aac(6′)-lb-cr,* *oqxA,* *oqxB*	*fosA, dfrA12* *tet(A)*	*mrkABCDFHIJ* *ybtEPQSTU*	*acrA, marAR, soxSR, ramAR, rob, sdiA, fis, envR, oqxAR, rarA*	*wzcwzi*
A18940/21	512	IncFIB(K) IncFII(K) IncN IncX3	*bla*_SHV-182_ *bla*_OXA-10_	*bla*_KPC-2_, *bla*_NDM-1_	*aac(6′)-Ib-cr,* *aac(6′)-Ib,* *aph(3′)-Ia,* *aadA2,* *aadA16*	*qnrS1,* *aac(6′)-lb-cr, oqxA,* *oqxB*	*fosA, sul1,* *dfrA12, dfrA27* *catA1*	*iutA, mrkABCHI*	*acrR*, *marAR* *soxRS, ramA, rob, sdiA, fis, envR, oqxR, rarA*	*wzi*
A1746/22	35	IncC, IncR, IncFIA(HI1) IncFIB(K) IncFIB (pKPHS1) IncFIB(pQil) IncFII(K)	*bla*_SHV-33_ *bla*_OXA-10_ *bla*_TEM-1B_ *bla*_VEB-25_ *bla*_DHA-1_	*bla*_KPC-2_	*ant(2* *″)-Ia, aph(3* *″)-Ib aph(6)-Id, rmtB,* *aadA1*	*qnrB4* *oqxA,* *oqxB*	*fosA, sul1* *sul2, catA1,* *cmlA1, tet(A), tet(G)*	*kfuA, mrkAFHI,* *ybtEQTX*	*acrR*, *marAR, soxRS, ramA* *rob, sdiA*, *fis, envR, oqxR rarA*	*wzc, wzi*
A7213/22	11	ColRNAI IncC IncFIA(HI1) IncFIB(K)	*bla*_OXA-1_ *bla*_OXA-10_ *bla*_CTX-M -15_ *bla*_TEM-1B_ *bla*_VEB-1_	*bla*_NDM-1_	*aac(6* *′* *)-Ib,* *aac(3)-IIa,* *aac(6′)-Ib-cr,* *ant(2″)-Ia,* *aph(3′)-Ia,* *rmtB*	*aac(6′)-lb-cr, oqxA, oqxB*	*fosA, sul2, dfrA14,* *tet(A)*	*mrkAC*	*soxSR, ramAR,* *sdiA*	*wzi*
A9974/22	11	ColRNAI IncC IncFIA(HI1) IncFIB(K) IncFII(K)	*bla*_SHV-159_ *bla*_OXA-1_ *bla*_OXA-10_ *bla*_CTX-M-15_ *bla*_TEM-1B_ *bla*_VEB-1_	*bla*_NDM-1_	*aac(6′)-Ib-cr* *aac(6′)-Ib* *aac(3)-IIa* *aph(6)-Id* *aph(3′)-Ia* *aph(3″)-Ib* *ant(2* *″* *)-Ia* *aadA1, rmtB*	*aac(6′)-lb-cr, oqxA, oqxB*	*fosA, sul2, dfrA14, tet(A), tet(G)*	*fyuA, mrkABCDFHIJ,* *ybtAEPQST*	*acrA, marAR soxR, ramAR* *rob, sdiA, fis*, *envR, oqxAB*, *rarA*	*wzi*
A10037/22	15	IncC IncFIA(HI1) IncFIB(K) IncFII(K)	*bla*_SHV-100_ *bla*_OXA-1_ *bla*_OXA-10_ *bla*_CTX-M-15_ *bla*_TEM-1B_ *bla*_VEB-1_	*bla*_NDM-1_	aac(6′)-Ib-cr *aac(6′)-Ib* *aac(3)-IIa* *aph(3″)-Ib* *aph(6)-Id* *ant(2″)-Ia* *aadA1, rmtB*	*aac(6′)-lb-cr, oqxA,* *oqxB*	*fosA, sul2* *dfrA14, cmlA1, tet(A), tet(G)*	*fyuA, kfuAC,* *mrkABCFHJ, ybtAEPQSTU*	*acrAR, marAR* *soxSR, rob, sdiA* *fis*, *envR, oqxAR* *rarA*	*wzi*
A10143/22	11	ColRNAI IncFII(K)	*bla*_OXA-1_ *bla*_OXA-10_ *bla*_CTX-M-15_ *bla*_TEM-1B_ *bla*_VEB-1_	*bla*_NDM-1_	*aac(6′)-Ib-cr* *aac(6′)-Ib* *aac(3)-IIa* *aph(3″)-Ib* *aph(6)-Id* *ant(2″)-Ia* *aadA1, rmtB*	*aac(6′)-lb-cr, oqxA, oqxB*	*fosA, sul1, sul2, dfrA12, dfrA14, cmlA1,* *tet(A), tet(G)*	*fyuA, mrkH,* *ybtEPQSX*	*marA, soxSR, ramR, sdiA,* *fis*, *envR*	*wzi*

Yersiniabactin cluster: *ybt*, *irp,* and *fyuA* genes; Aerobactin cluster: *iuc* and *iut* genes; AcrAB efflux pump: *acr, mar, sox, rob*, ram *sdiA, fis, and envR,* genes; OqxAB efflux pump: *oqx* and *rar* genes.

**Table 2:** Clinical data of carbapenem-resistant *Klebsiella pneumoniae* isolates.

Strain	Date of Isolation	Sex	Age (Years)	Ward	Biological Sample	Infection/Colonization
C2482/18	9 June 2018	F	8	PICU	Rectal swab	Colonization
C4112/18	7 September 2018	F	56	ICU	Rectal swab	Colonization
B11395/18	24 October 2018	F	1	PICU	Urine	Infection
D6184/18	19 November 2018	F	8	PICU	Bronchial aspirate	Infection
C251/19	17 January 2019	F	17	PICU	Rectal swab	Colonization
Z557/19	30 January 2019	M	57	ICU	Rectal swab	Colonization
C746/19	7 February 2019	M	73	ICU	Rectal swab	Colonization
B2562/19	4 March 2019	M	79	ICU	Urine	Infection
Z508/19	20 March 2019	F	60	ICU	Rectal swab	Colonization
D1463/19	22 March 2019	M	55	ICU	Bronchial aspirate	Infection
D1598/19	1 April 2019	M	40	ICU	Bronchial aspirate	Infection
Z852/19	12 April 2019	M	3	PICU	Rectal swab	Colonization
Z866/19	15 April 2019	M	49	ICU	Rectal swab	Colonization
D2452/19	24 May 2019	M	73	ICU	Bronchial aspirate	Infection
C833/21	31 March 2021	F	57	ICU	Wound	Infection
A5051/21	25 April 2021	F	69	ICU	Blood	Infection
D2856/21	7 July 2021	F	54	ICU	Bronchial aspirate	Infection
C1909/21	8 July 2021	M	85	ICU	Wound	Infection
A18940/21	24 December 2021	F	58	ICU	Blood	Infection
A1746/22	25 February 2022	M	0.25	PICU	Blood	Infection
A7213/22	3 May 2022	F	30	ICU	Blood	Infection
A9974/22	16 June 2022	F	67	ICU	Blood	Infection
A10037/22	16 June 2022	F	53	ICU	Blood	Infection
A10143/22	19 June 2022	M	88	ICU	Blood	Infection

ICU: Intensive Care Unit, PICU: Pediatric Intensive Care Unit.

## Data Availability

The data of this study have been deposited in the European Nucleotide Archive (ENA) at EMBL-EBI under accession numbers PRJEB42192 and PRJEB46676.
